# Does colectomy affect the progression of primary sclerosing cholangitis? A systematic review and meta-analysis 

**Published:** 2018

**Authors:** John Ong, Michael F. Bath, Carla Swift, Yasseen Al-Naeeb

**Affiliations:** 1 *Department of Engineering, University of Cambridge, Trumpington Street, Cambridge CB2 1PZ, UK*; 2 *Department of Gastroenterology, Bedford Hospital South Wing, Kempston Road, Bedford MK42 9DJ, United Kingdom*; 3 *Department of Surgery, Addenbrooke's Cambridge University Hospital, Hills Road, CB2 0QQ, United Kingdom*; 4 *Department of Gastroenterology, Addenbrooke's Cambridge University Hospital, Hills Road, CB2 0QQ, United Kingdom*

**Keywords:** Primary sclerosing cholangitis, Inflammatory bowel disease, Colectomy, Procto-colectomy

## Abstract

**Aim:**

The aim of this systematic review was to determine if the human colon, through the lower gut-liver axis, drives PSC activity by assessing the progression of the disease in patients with and without colectomy for colonic disease.

**Background:**

The gut-liver axis is involved in the pathogenesis of liver disease. Abnormal immune-mediated responses to intestinal microbiome are implicated in primary sclerosing cholangitis (PSC) however the mechanisms remain poorly understood. Currently, no single animal model recapitulates all attributes of PSC in humans and this limits further studies of gut-liver interactions.

**Methods:**

A systematic search of PubMed, Medline, and Scopus was performed for articles that contained the terms “colectomy” or “bowel resection” AND “primary sclerosing cholangitis” up to 15th April 2018. Articles were reviewed by 2 reviewers and raw data collated. A Forest plot was used to illustrate the effect of colectomy on subsequent liver transplantation for PSC. Linear regression was used to estimate mortality risk.

**Results:**

Colectomy appeared to have no effect on PSC progression, although high-quality studies were lacking. Rates of liver transplantation or transjugular intrahepatic portosystemic shunt for PSC were not affected by colectomy (OR 0.59, 95% CI 0.14 - 2.53, p=0.48). Mortality risk following colectomy in patients with PSC is 2.11% per year (95% CI 0.03% - 4.18%, p=0.032, R2 = 0.722).

**Conclusion:**

Current evidence is limited but suggests colectomy does not affect the progression of PSC in patients with colonic disease. Pathogenic micro-organisms or antigens that drive PSC may not be limited to the lower gut.

## Introduction

 The gut-liver axis, a concept thought to be first introduced in 1987 by Volta et al (1-2), describes the complex interactions between the gut microbiome, the small and large bowel, the immune system and the liver. Recent advances in gastroenterology have implicated the gut-liver axis in alcoholic liver disease, non-alcoholic fatty liver disease, primary biliary cholangitis, and primary sclerosing cholangitis (PSC) (3). In PSC, immune-mediated processes lead to chronic inflammation of both intra-hepatic and extra-hepatic bile ducts, eventually causing liver cirrhosis. Approximately 47-76% of patients with PSC also suffer from inflammatory bowel disease (IBD) (4), however, a causal link has not been established. Dysbiosis in the gut microbiome, increased intestinal permeability, translocation of circulating pro-inflammatory cytokines in the portal vein, and circulating auto-antibodies are hallmark characteristics in the pathophysiology of PSC (3), however little else is known about the triggering microorganism, relevant antigens, or its location within the gastrointestinal tract. 

The role of the colon in the pathogenesis of PSC has long served as a controversial point of debate. Early small sample sized case series (5-7) have previously reported impressive rates of improvement in PSC for patients who had undergone colectomy for concomitant ulcerative colitis (UC). However, such results were irreproducible in larger cohort studies of UC patients (8-9). In support of gut involvement in PSC, basic science research has elucidated the recruitment and preferential binding of chemokine (C-C motif) receptor 9 positive - integrin α_4_β_7_ positive (CCR9+ α_4_β_7_+) T-lymphocytes to abnormally upregulated mucosal vascular addressin cell adhesion molecule 1 (MAdCAM-1), vascular adhesion protein-1 (VAP-1) and gut homing chemokine (C-C motif) ligand (CCL25) on hepatic endothelial cells, and the up-regulation of toll-like receptors on biliary epithelial cells and T-Helper type 17 (TH17) cells as responses of the gut-liver axis specific to PSC (10-13). Clinically, a retrospective study from a transplant centre has also demonstrated that colectomy conferred a protective effect to liver grafts against recurrent PSC (14). Such conflicting reports in literature preclude any strong conclusions from being made. 

In looking at animal models to understand the role of the colon in the pathogenesis of PSC, current disease models of PSC remain sub-optimal and not a single animal model to date is able to fully recapitulate all attributes of the disease seen in humans (15). As a result, further studies of gut-liver interactions are restricted and this impacts on our understanding of the disease and our ability to develop potential treatments. In order to determine if the lower gut-liver axis (colon-microbiome-immune interactions) drives the disease activity in PSC, the aim of this systematic review was to determine if total colectomy had any effect on the progression of PSC. 

## Methods

**Literature search**

The systematic review was performed following standard PRISMA guidelines (16). A search of Medline, Cochrane, and Scopus databases was performed for articles containing the terms "colectomy" AND "primary sclerosing cholangitis" or “bowel resection” AND "primary sclerosing cholangitis" that were published up to April 2018. Unpublished literature was identified through the OpenGrey database. Additional studies that were not included in the database search were identified through searching the reference lists of retained articles.

**Inclusion and exclusion criteria**

All observational studies, except for case reports and small case series (less than five patients), were included for further evaluation when available. All patients with a confirmed diagnosis of PSC were included. Patients who had PSC but not undergone colectomy were used as a control group, and total colectomy or procto-colectomy were considered as an identical intervention, where applicable. The exclusion criteria for meta-analyses were: (i) studies that did not report sufficient primary data, (ii) studies with irrelevant content and (iii) studies that were not accessible by the UK Access Management Federation.

**Data extraction**

Two authors (JO and MFB) independently reviewed all titles and abstracts then assessed articles against the inclusion criteria for analysis. A third reviewer (YAN) resolved any differences. The primary outcome measure was the progression of PSC as determined by serological or histological evidence, defined pragmatically on the specific criteria used within each study. Secondary outcome measures were the rate of liver transplantation (including re-transplantation) or transjugular intrahepatic portosystemic shunts (TIPSS) when liver transplantation was unsuitable, and mortality rates. Data were extracted independently by authors JO and MFB using a standardized form. Data extracted included serological or histological progression of PSC, transplantation rates, and mortality rates. Quality of the studies included was assessed using a modified Newcastle–Ottawa Scale.

**Table 1:** PSC progression rates in patients with colonic disease, with and without colectomy

Study, Year	Type of Resection	Follow-up (years)	Sample size in study		No change to PSC activity or PSC progression	
			Colectomy, n	No Colectomy, n	Colectomy, n (%)	No Colectomy, n (%)
Cangemi et al, 1989 (8)	PC	3	13	17	13 (100%)	17 (100%)
Alabraba et al,2009 (14)	PC or CO	6.9	46	169	15 (32.6%)	39 (23.1%)
Aitola et al, 1994 (17)	PC or CO	4.8	7		5 (71.4%)	
Mikkola et al, 1995 (18)	PC or Co	9	13		4 (30.8%)	
Goudet et al, 2000 (9)	PC or CIA	10	36		18 (50.0%)	
Cho et al, 2008 (19)	IPA	4.3	22		2 (9.1%)	
Lepisto et al, 2009 (20)	PC, IPA	11	30		15 (50%)	

PC = Proctocolectomy, Co = Colectomy, IPA = Ileal pouch-anal canal anastomosis, CIA = Colectomy with ileorectal anastomosis,

* = no control group in study

**Table 2:** Liver transplantation or TIPSS rates in patients with colonic disease, with and without colectomy

Study, Year	Type of Resection	Follow-up (years)	Sample size in study		Transplantation or TIPSS	
			Colectomy, n	No Colectomy, n	Colectomy, n (%)	No Colectomy, n (%)
Poritz et al, 2003 (21)	PC or Co	7	16	6	6 (37.5%)	*
Navaneethan et al, 2011 (22)	PC, PBI, or IPA	13.4	92	75	30 (32.6%)	56 (74.7%)
Nordenvall et al, 2018 (23)	Co or SR	5.9	477	2092	51 (10.7%)	276 (13.2%)
Lepisto et al, 2009 (20)	PC	11	30		15 (50%)	
Mathis et al, 2012 (24)	PC	5.9	100		9 (9%)	
Lian et al, 2012 (25)	Co	15	23		9 (39.1%)	

*OLTx = Liver Transplant, PC = Proctocolectomy, Co = Colectomy, SR = Segmental large bowel resection*,* IPA = Ileal pouch-anal canal anastomosis, PBI = proctocolectomy with Brooke’s ileostomy, *

* *= no control group in study*

**Data synthesis and analysis**

Forest plots were used to illustrate effect size between studies, where possible. Each study included had an odds ratio (OR) with respective 95 percent confidence interval (CI) calculated. A Mantel-Haenszel (M-H) statistical method was performed, calculating an overall OR for respective outcomes: an OR less than 1.00 inferred a worse survival for the colectomy group versus the control group, whilst an OR greater than 1.00 inferred a better survival. The significance level was set to 5% for all tests and alternative hypotheses were two-sided. High heterogeneity between studies was presumed and a random-effects model was used. A funnel plot was employed to assess for potential publication bias (Appendix 1). 

All-cause mortality following colectomy was plotted for each study against respective mean study follow up. A mean-weighted linear regression was performed to estimate the change in all-cause mortality per year.

Statistical analysis was performed using Review Manager (RevMan) Version 5.3 (Copenhagen: The Nordic Cochrane Centre, The Cochrane Collaboration, 2014) and GraphPad Prism 5.0 (GraphPad Software, La Jolla California USA).

## Results

**Figure 1:**
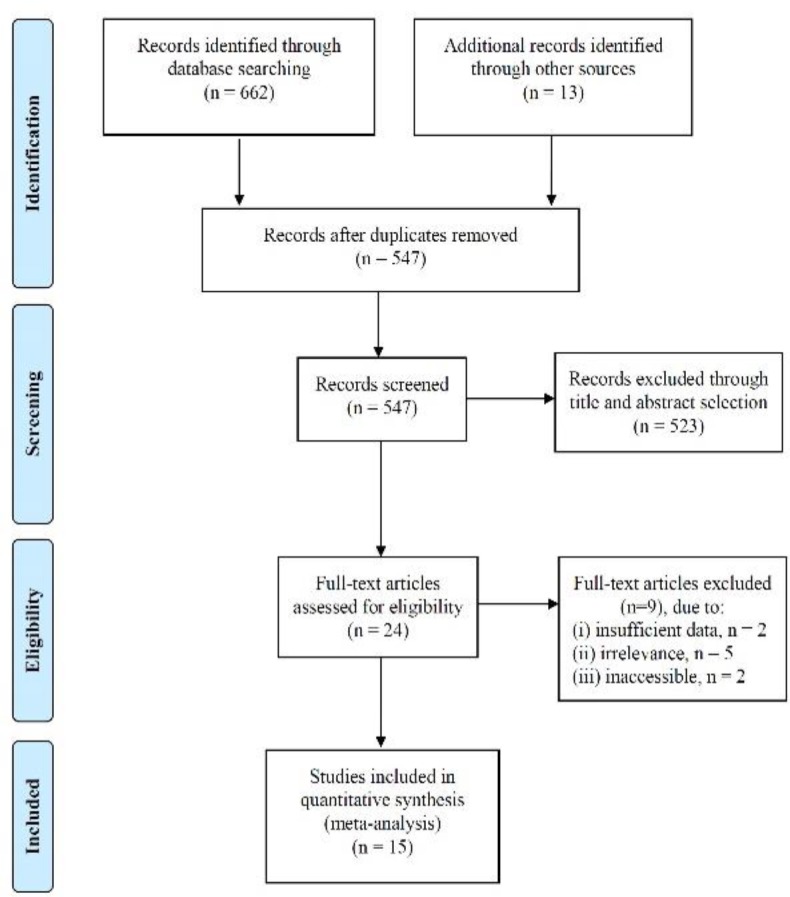
Systematic review flow diagram

675 studies were identified from the initial literature search (Figure 1). Following removal of duplicates and abstract screening, twenty-four full-text articles were reviewed. After excluding articles with insufficient data (n=2), irrelevant content (n=5) and inaccessibility (n=2), a total of fifteen articles were included in the final review (8-28).

**Rate of PSC Progression**

Overall, colectomy did not appear to have an effect on PSC progression. Two studies directly comparing patients with PSC following colectomy versus no colectomy were identified (Table 1) but there was no significant difference between both groups of patients. No randomized control trials were identified; the limited number of studies identified precluded additional quantitative analysis.

**Figure 2:**
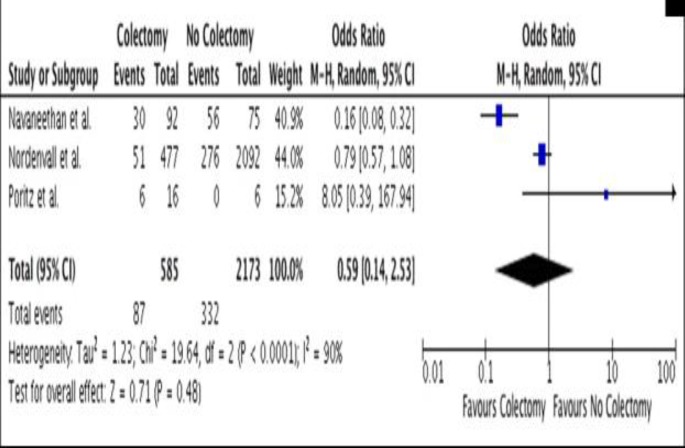
Forest Plot-Effect of colectomy on liver transplantation of TIPSS in PSC patients

**Table 3:** Studies reporting mortality rates in PSC patients with colonic disease, with and without colectomy

Study, Year	Type of Resection	Follow-up (years)	Sample size in study		Mortality Rates	
			Colectomy, n	No Colectomy, n	Colectomy, n (%)	No Colectomy, n (%)
Cangemi et al, 1989 [8]	PC	3	13	17	2 (15.3%)	2 (11.8%)
Nordenvall et al, 2018 [23]	Co	5.9	477	2092	83 (17.4%)	426 (20.4%)
Post et al, 1994 [26]	PC, Co	0.1	24		3 (12.5%)	
Penna et al, 1996 [27]	IPA	4.5	54		6 (11.1%)	
Gorgun et al, 2005 [28]	IPA	5	65		16 (24.6%)	
Lian et al, 2012 [25]	Co	15	23		8 (34.8%)	
Treeprasertsuk et al, 2013 29]	Co	5.5	78		13 (16.7%)	

*PC = Proctocolectomy, Co = Colectomy, IPA = Ileal pouch-anal canal anastomosis, *

* *= no control group in study*

**Figure 3:**
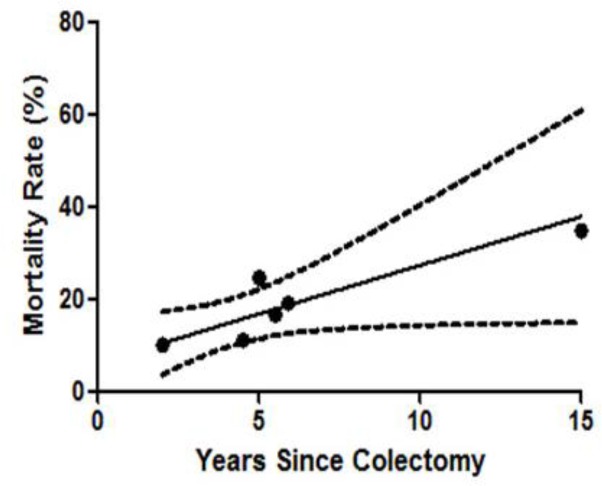
Linear regression of mortality following colectomy; gradient 2.11, p= 0.032 R^2^=0.722

One study (8) followed up 30 patients in total over 3 years follow-up, reporting disease progression in all patients, both colectomy and non-colectomy. Another study (14) reported data on 46 patients who had undergone a colectomy and 169 patients as the control, for an average follow-up of 6.9 years, and noted PSC progression rates as 32.6% and 23.1% respectively (p=0.19).

Five further observational studies were identified with a follow-up period ranging between 4.3 to 11 years (9, 17-20). However, these studies did not have control groups so statistical comparisons were not possible. The reported PSC progression rates ranged between 9.1% and 50%. Due to heterogeneity in the definitions of PSC progression used in the studies, linear regression analysis was not attempted.

**Rates of Liver Transplantation or TIPSS**

Six studies (20-25) reporting liver transplantation rates (or TIPSS in cases where liver transplantation was unsuitable) were included (Table 2). Follow-up ranged from 5.9 to 13.4 years, with rates reported between 9% and 50%. Three studies (21-23) directly compared colectomy versus no colectomy in this patient cohort. Following forest plot analysis, no significant effect of colectomy on either the rates of liver transplantation or TIPSS was demonstrated (OR 0.59, 95% CI 0.14-2.53, p=0.48) (Figure 2). 

**All-Cause Mortality Rates**

Seven studies were identified that reported all-cause mortality rates following colectomy (8, 23, 25-29) (Table 3). Mean-weighted linear regression analysis (Figure 3) demonstrated a 2.11% per year mortality risk (CI 0.03% to 4.18%, p=0.032 R^2^ = 0.722) for patients with PSC who have undergone colectomy.

Two studies (8, 23) directly compared colectomy versus no colectomy group in PSC patients. One study (8) followed up 30 patients for 3 years and demonstrated similar mortality between the two groups, with rates of 15.3% and 11.8% respectively. The other study (23) identified, followed 2569 patients for a median time of 5.9 years, and also showed no difference in mortality rates for all time points of colectomy, reporting 17.4% versus 20.4% respectively.

## Discussion

Our results suggest that cumulatively there is limited evidence in the current literature to demonstrate any beneficial effect of colectomy on the disease activity of PSC. Nordenvall et al (23) reported that colectomy before the diagnosis of PSC was associated with lower liver transplantation and death rates in IBD-PSC patients and no effect was observed if colectomy was performed after PSC was diagnosed. Though interesting, these findings contradict a study by Alabraba et al (14) which reported that colectomy before or during liver transplantation for PSC significantly reduced PSC recurrence and liver re-transplantation rates. However, both the numbers in these specific subgroups were sub-optimal. Taking into consideration that there is a lack of an established scientific mechanism through which these effects are achieved, an argument that these observations are due to chance alone could also be made.

We were able to calculate an estimated mortality rate, based on the included studies, for patients with PSC following colectomy at 2.11% per year. Putting this into context, mortality rates for all PSC patients reported in the literature ranges between 3.3-5.8% (35-39), and whilst our data may therefore suggest a lower mortality rate post-colectomy, the heterogeneity and low quality in the studies included precludes any definitive conclusions to be drawn. In considering the above, together with the lack of robust evidence, we believe colectomy should not be offered as a treatment option for severe PSC until better patient studies or scientific advances are able to demonstrate otherwise.

The main limitation of this systematic review was the lack of high-quality studies for meta-analyses in current literature. No randomized control trials were identified and only a small proportion of studies had a control group so that an effect size could be estimated. Another limitation was the high heterogeneity between studies, which was in part due to varying criteria of how disease progression was measured and defined within these studies. In this regard, it precluded meaningful quantitative analyses in this systematic review. Lastly, though the statistical analyses performed herein were robust, effects from small sample sizes in the meta-analyses and publication bias cannot be excluded completely. Nonetheless, the main aim of this systematic review was to study PSC activity after the interference of the lower gut-liver axis is achieved through a "surgical knock-out" of the colon in humans. 

As animal models remain sub-optimal, observational studies such as case-control and association studies, although do not demonstrate causality, still provide important information and have contributed too much of our understanding of PSC (3). However, even in reviewing clinical studies of PSC in recent literature, it seems the role of the colon in the pathogenesis of PSC remains poorly understood and the chasm between basic sciences and clinical observations remain wide and poorly bridged. Invariably, this is reflected in the limited treatment options in clinical practice. It is also noteworthy that although well designed longitudinal studies could offer alternative means to identify causative microorganisms, the lack of a reliable biomarker in early disease, low prevalence of the disease, and poor accessibility of the biliary tree restrict the conduct of these studies (15). 

Moving away from animal models and clinical observational studies, in vitro longitudinal and high throughput screens of the microbiome in the lower GI tract offers the possibility of identifying the pathogenic microorganisms in PSC as the cost of interrogating the microbiome becomes more affordable. Unfortunately, even if a pathogen is identified, the transition from mechanistic studies to identify a target for drug action and then to drug safety studies preclude the use of any pharmacological treatments in the near future. In considering novel and potential treatments for PSC that are on the horizon, faecal transplantation is perhaps the closest at being introduced into clinical practice. Fecal transplantation could potentially reverse gut dysbiosis in PSC and provide a means of controlling PSC progression where current drug treatments have failed. Interestingly, a clinical trial assessing the effects of faecal transplantation in patients with PSC is currently in progress (ClinicalTrials.gov, Identifier: NCT02424175) and the results of this study are eagerly awaited. Alternatively, advances in regenerative medicine (40,41) have made considerable progress in our understanding of cholangiocyte biology. In vivo studies and transplantation of lab-grown bile ducts are being undertaken in porcine models which could potentially be used in the treatment of large duct PSC (University of Cambridge, UK). 

In summary, the colon as part of the lower gut-liver axis is likely to be involved in the pathogenesis of PSC but is unlikely to be the key factor driving the disease and higher-quality larger clinical trials are required. The pathogenic microbiome or antigens could potentially be identified in the midgut, since total colectomy has shown little effect on PSC activity. Though our understanding of the immune-mediated disease remains poor, rapid advances in regenerative medicine, high throughput screening of the gut microbiome, and research targeting the gut-liver axis are likely to make headway in developing new treatments for the disease.

**Appendix 1:**
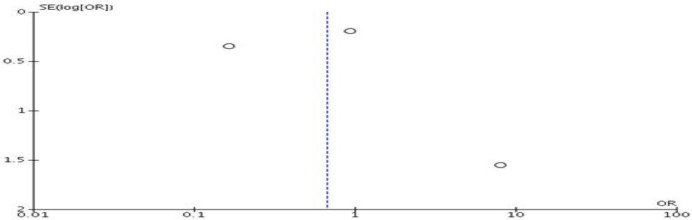
Funnel plot for studies induced in liver transplantation and TIPSS rate
